# Multi-Omics analysis and in vitro validation reveal diagnostic and therapeutic roles of novel hub genes in ovarian cancer

**DOI:** 10.1186/s41065-025-00535-z

**Published:** 2025-08-18

**Authors:** Jianmin Wang, Guanghui Song, Lili Xing

**Affiliations:** 1 Department of Obstetrics and Gynecology, Sir Run Run Shaw Hospital, Key Laboratory of Reproductive Dysfunction Management of Zhejiang Province, Zhejiang University School of Medicine, Hangzhou, Zhejiang 310016 China; 2 Center for Reproductive Medicine, Department of Reproductive Endocrinology, Zhejiang Provincial People’s Hospital, Hangzhou Medical College, Hangzhou, 310014 China

**Keywords:** OC, Hub genes, Biomarker, Therapeutic target

## Abstract

Ovarian cancer (OC) remains a highly lethal gynecologic malignancy due to late diagnosis and limited therapeutic options. In this study, we aimed to identify and functionally validate novel hub genes associated with OC progression. We integrated four GEO microarray datasets (GSE54388, GSE40595, GSE18521, and GSE12470) to identify differentially expressed genes (DEGs) between OC and healthy tissues using the limma package. A total of 22 common DEGs were identified, of which four—SNRPA1, LSM4, TMED10, and PROM2—emerged as hub genes based on PPI network centrality. Expression analyses using TCGA data and RT-qPCR confirmed the significant upregulation of these genes in OC samples. Promoter methylation analysis showed hypomethylation in tumors, while ROC analysis revealed high diagnostic accuracy (AUC = 1.0). Although these genes were not significantly associated with overall survival in meta-analysis, they were strongly involved in oncogenic pathways such as EMT, apoptosis, and DNA repair. Predicted miRNAs (e.g., hsa-miR-1178-5p and hsa-miR-31-5p) targeting hub genes were significantly downregulated in OC cell lines. Immune analysis indicated that hub gene expression was correlated with immune subtypes, checkpoint inhibitors, and reduced immune infiltration. Drug sensitivity analysis suggested that high expression of TMED10 and PROM2 may confer susceptibility to chemotherapeutic agents. Functional assays following siRNA-mediated knockdown of TMED10 and PROM2 in A2780 and OVCAR3 cells revealed significant reductions in proliferation, colony formation, and migration. These findings highlight SNRPA1, LSM4, TMED10, and PROM2 as potential diagnostic markers and therapeutic targets in OC, warranting further investigation for clinical translation.

**Supplementary Information:**

The online version contains supplementary material available at 10.1186/s41065-025-00535-z.

## Introduction

Ovarian cancer (OC) remains one of the most lethal gynecologic malignancies worldwide, characterized by its asymptomatic progression, late-stage diagnosis, and high rates of recurrence and chemoresistance [1, 2]. According to the Global Cancer Statistics 2024, OC is the eighth most commonly diagnosed cancer and the fifth leading cause of cancer-related deaths among women, accounting for over 313,000 new cases and 207,000 deaths annually [3, 4]. The five-year survival rate remains below 45% for most patients diagnosed at advanced stages, highlighting the urgent need for earlier detection and more effective treatment strategies [5, 6].

Despite advances in imaging technologies, surgical techniques, and systemic therapies, the overall survival of OC patients has only modestly improved in recent decades [7, 8]. This stagnation is largely due to the molecular heterogeneity of ovarian tumors and the lack of reliable biomarkers for early diagnosis, prognosis, and treatment response [9, 10]. Therefore, identifying robust molecular targets and understanding the underlying gene regulatory networks involved in OC progression are essential for developing precision medicine strategies.

In recent years, high-throughput expression profiling and bioinformatics analyses have revolutionized cancer research by enabling the identification of differentially expressed genes (DEGs), molecular signatures, and key regulatory pathways associated with various malignancies, including OC [11, 12]. Several studies have utilized publicly available datasets such as the Gene Expression Omnibus (GEO), The Cancer Genome Atlas (TCGA), and ArrayExpress to screen for potential diagnostic, prognostic, or therapeutic biomarkers in OC. For instance, Chu et al. employed GEO datasets and protein–protein interaction (PPI) network analysis to identify potential biomarkers such as COL1A1 and THBS1, which are implicated in extracellular matrix remodeling and tumor invasion [13]. Similarly, Wu et al. used integrative bioinformatics approaches combining DEG analysis, functional enrichment, and survival analysis to identify FN1, ITGB1, and MMP9 as key genes linked to OC metastasis and poor prognosis [14].

While these studies represent significant progress in the field, several important limitations persist. Many investigations rely exclusively on in silico approaches without validating the findings in biological models, which raises concerns about the reproducibility and clinical relevance of the proposed biomarkers. Moreover, due to differences in patient populations, platforms, and experimental conditions, findings from individual datasets may lack generalizability when not validated across independent cohorts or through experimental replication. Some studies also focus on identifying DEGs without further investigating their functional roles in tumorigenesis, such as cell proliferation, apoptosis, and migration, or therapeutic response [15, 16].

Additionally, most previous works have not incorporated multi-omics integration, such as combining transcriptomic data with methylation, mutational landscape, immune cell infiltration, or non-coding RNA regulation, which are increasingly recognized as critical in understanding the complex biology of OC. The absence of wet-lab validation, such as RT-qPCR, Western blotting, gene knockdown, or rescue experiments in relevant OC cell lines, further limits the translational impact of these findings.

Therefore, there is a growing need for studies that bridge computational predictions [17, 18] with experimental validation [19, 20] to establish a clearer understanding of gene function and clinical applicability. Our study addresses this critical gap by employing a comprehensive pipeline that integrates GEO-based transcriptomic profiling with in vitro validation of key hub genes in OC cell lines, thereby enhancing the robustness, reliability, and translational potential of the results.

## Methodology

### Microarray dataset selection and preprocessing

Four publicly available gene expression datasets related to OC were retrieved from the Gene Expression Omnibus (GEO) database [21]: GSE54388, GSE40595, GSE18521, and GSE12470. These datasets were selected based on the following inclusion criteria: (i) datasets comprising both OC and healthy ovarian tissue samples; (ii) profiling conducted on human samples; and (iii) availability of raw or processed expression data. The datasets were downloaded from the GEO repository (https://www.ncbi.nlm.nih.gov/geo/), and platform annotation files were used to map probe IDs to official gene symbols.

### Differential expression analysis

Differential gene expression analysis between OC and healthy ovarian tissue samples was performed independently for each dataset using the limma (Linear Models for Microarray Data) package (v3.52.2) in R (v4.2.0) (https://www.r-project.org/). Expression data were log2-transformed and normalized using quantile normalization. Linear modeling and empirical Bayes moderation were applied to obtain moderated t-statistics, log2 fold changes (log2FC), and adjusted p-values using the Benjamini–Hochberg false discovery rate (FDR) correction. Genes with an adjusted *p* < 0.05 were considered statistically significant. Volcano plots were generated using the ggplot2 package (https://ggplot2.tidyverse.org/) to visualize the distribution of differentially expressed genes (DEGs) in each dataset.

### Integration of DEGs across datasets

To identify robust and consistently dysregulated genes across datasets, lists of top 2000 significant DEGs from each dataset were intersected using the VennDiagram package in R (https://cran.r-project.org/web/packages/VennDiagram/).

### Identification of hub genes

Common DEGs were subjected to protein–protein interaction (PPI) network analysis using the STRING database (v11.5) (https://string-db.org/) [22] with a minimum interaction confidence score of 0.7. The resulting PPI network was imported into Cytoscape software (v3.9.1) (https://cytoscape.org/) for visualization and topological analysis. Node degree centrality was used to identify highly connected genes. Hub genes were selected based on a combination of high connectivity within the network.

### Cell culture

Nine human OC cell lines (A2780 [ECACC 93112519], OVCAR3 [ATCC^®^ HTB-161™], SKOV3 [ATCC^®^ HTB-77™], CAOV3 [ATCC^®^ HTB-75™], SW626 [ATCC^®^ HTB-78™], ES2 [ATCC^®^ CRL-1978™], OVCAR4 [ATCC^®^ NCI-DTP OVCAR-4™], OV90 [ATCC^®^ CRL-11732™], and COAV (ATCC^®^ HTB-75™]) were purchased from ECACCC and ATCC USA. Five healthy human ovarian epithelial control cell lines, including HOSEpiC (ScienCell, Cat. #7310), HOEpiC (ATCC, Cat. #PCS-460-010), hOSEC (Innoprot, Cat. #4810), and HOVEC (PromoCell, Cat. #C-12260) were acquired from other certified vendors. All cancer and healthy ovarian cell lines were cultured under standard conditions recommended by their respective suppliers. A2780, OVCAR3, SKOV3, and CAOV3 ovarian cancer cell lines were maintained in RPMI-1640 medium (Thermo Fisher Scientific, Cat# 11875093) supplemented with 10% fetal bovine serum (FBS) (Thermo Fisher Scientific, Cat# 16000044) and 1% penicillin-streptomycin (Thermo Fisher Scientific, Cat# 15140122). IGROV1, TOV112D, OV90, and Kuramochi cells were cultured in either DMEM (Thermo Fisher Scientific, Cat# 11965092) or MCDB105/M199-based media supplemented with 10% FBS and antibiotics, as per cell line-specific guidelines. ES2 cells were cultured in McCoy’s 5 A medium (Thermo Fisher Scientific, Cat# 16600082) with 10% FBS and 1% penicillin-streptomycin. Healthy ovarian epithelial cell lines (HOSEpiC, IOSE80, IOSE397, T1074, and NOV-31) were grown in Ovarian Epithelial Cell Medium (OEpiCM) from ScienCell Research Laboratories (Carlsbad, CA), supplemented with epithelial growth supplements and 5% FBS. All cell lines were maintained in a humidified incubator at 37 °C with 5% CO₂, and cells were harvested for downstream assays at 70–80% confluency.

### Real time quantitative PCR (RT-qPCR) analysis

Total RNA was extracted using TRIzol reagent (Invitrogen, USA) according to the manufacturer’s instructions [23]. Reverse transcription was performed using the RevertAid First Strand cDNA Synthesis Kit (Thermo Fisher Scientific, USA). RT-qPCR was carried out using SYBR Green Master Mix (Applied Biosystems, USA) on a QuantStudio 6 Flex Real-Time PCR System. GAPDH served as the internal control. Gene-specific primers were used to assess the expression levels of SNRPA1, LSM4, TMED10, and PROM2. Relative quantification was performed using the 2^^−ΔΔCt^ method. All assays were conducted in biological triplicates. Following primers were used in RT-qPCR assay.

GAPDH-F 5’-ACCCACTCCTCCACCTTTGAC-3’,

GAPDH-R 5’-CTGTTGCTGTAGCCAAATTCG-3’.

SNRPA1-F: 5’-ATCCAGGTGCTGGTTTGCCAAC-3’.

SNRPA1-R: 5’-GGATCTGACCAGACTGCAGCAA-3’.

LSM4-F: 5’-GGAGACGTACAATGGACACCTG-3’.

LSM4-R: 5’-ATGCGCAGGTACTTGATGGTGC-3’.

TMED10-F: 5’-CTCCAAAGAGGATGCAACCAAGG-3’.

TMED10-R: 5’-TCACGAGTTGGTCAGGTATCCG-3’.

PROM2-F: 5’-CATCAGCATCCACCAAGCCTATC-3’.

PROM2-R: 5’-TGCAACTCCTGCCGTAGCTTGT-3’.

### Validation of hub gene expression in TCGA and pathway analysis

RNA-seq expression data from the TCGA TCGA-OV cohort were analyzed using the GEPIA2 platform (http://gepia2.cancer-pku.cn/) [24], which integrates TCGA and GTEx data. Expression levels of hub genes were compared between tumor and healthy ovarian tissues using log2 (TPM + 1) normalized values. Functional pathway associations of the hub genes were evaluated using the GSCA platform (http://bioinfo.life.hust.edu.cn/GSCA/) [25]. Spearman correlations between gene expression and the activity scores of 15 oncogenic pathways were computed using TCGA-OC data.

### Promoter methylation analysis

Promoter methylation levels of hub genes were assessed using the GSCA platform (http://bioinfo.life.hust.edu.cn/GSCA/) [25], which incorporates TCGA methylation data. Beta values representing methylation intensity (ranging from 0 to 1) were compared between OC and healthy ovarian tissues.

### Meta-analysis of prognostic significance

To assess the prognostic value of hub genes, survival meta-analysis was performed using the GENT2 database (http://gent2.appex.kr/gent2/) [26], which compiles gene expression and clinical outcome data from multiple GEO datasets.

### Mutational and copy number variation analysis

Mutation profiles of hub genes were analyzed using the cBioPortal for Cancer Genomics (https://www.cbioportal.org/) [27] based on TCGA-OC dataset. CNV data for the hub genes were obtained from cBioPortal’s GISTIC 2.0-based analysis. CNVs were categorized into heterozygous amplifications (Hete. Amp.), heterozygous deletions (Hete. Del.), and other events.

### Protein–Protein interaction (PPI) network construction and gene enrichment analysis

PPI networks for each hub gene (SNRPA1, LSM4, TMED10, and PROM2) were constructed using the Pathway Commons database (https://www.pathwaycommons.org/) [28]. Gene Ontology (GO) and Kyoto Encyclopedia of Genes and Genomes (KEGG) enrichment analyses were performed using the DAVID functional annotation tool (https://david.ncifcrf.gov/) [29].

### miRNA–hub gene interaction analysis

Putative miRNAs targeting the 3′ untranslated regions (3′ UTRs) of the hub genes were predicted using the TargetScan database (https://www.targetscan.org/vert_80/) [30]. The top predicted miRNAs—hsa-miR-4795-3p (SNRPA1), hsa-miR-4488 (LSM4), hsa-miR-31-5p (TMED10), and hsa-miR-1178-5p (PROM2)—were selected based on target site context scores and conservation. Predicted binding positions were recorded for each miRNA–mRNA interaction.

To experimentally validate the expression of predicted miRNAs, RT-qPCR was performed across ovarian cancer and healthy control cell lines using the TaqMan™ Advanced miRNA cDNA Synthesis Kit and miRNA-specific TaqMan™ probes (Thermo Fisher Scientific, USA), according to the manufacturer’s protocol. U6 small nuclear RNA was used as the endogenous control for normalization. All reactions were carried out in biological triplicates.

### Immunological subtype and immune correlation analysis

To evaluate the immune relevance of the hub genes in OC, expression and immune association data were analyzed using the TISIDB database (http://cis.hku.hk/TISIDB/) [31], which integrates TCGA molecular and immunological datasets. Correlation heatmaps were generated to examine the relationships between hub gene expression and immune inhibitor genes using TISIDB database. Moreover, GSCA database was utilized for the immune infiltration analysis of hub genes in OC.

### Drug sensitivity analysis

Drug response data were retrieved from the Genomics of Drug Sensitivity in Cancer (GDSC) database (https://www.cancerrxgene.org/) [32]. Spearman correlation analysis was performed between gene expression and the half-maximal inhibitory concentration (IC_50_) values of various chemotherapeutic agents.

### SiRNA transfection and functional assays

For functional assays, TMED10 and PROM2 were selected because they emerged as top hub candidates from our integrated transcriptomic and bioinformatics analyses. To investigate the functional role of TMED10 and PROM2 in OC, siRNA-mediated knockdown was performed in A2780 and OVCAR3 cell lines. These two cell lines were selected based on their high endogenous expression of TMED10 and PROM2, as observed in preliminary screening across multiple OC cell lines, making them suitable models for functional validation. Cells were seeded in six-well plates at a density of 2 × 10⁵ cells per well and transfected with either TMED10- or PROM2-specific small interfering RNAs (siRNAs) or a negative control siRNA (Ctrl) using Lipofectamine RNAiMAX transfection reagent (Invitrogen, USA), following the manufacturer’s protocol. The final siRNA concentration was 50 nM. Knockdown efficiency was confirmed 48 h post-transfection via RT-qPCR and Western blotting [33, 34].

For Western blot analysis, cells were lysed using RIPA buffer supplemented with protease and phosphatase inhibitors (Thermo Fisher Scientific), and protein concentrations were determined using the Pierce™ BCA Protein Assay Kit. Equal amounts of protein (30 µg) were separated by SDS-PAGE and transferred to PVDF membranes (Thermo Fisher Scientific). Membranes were blocked with 5% non-fat milk in TBST and incubated overnight at 4 °C with primary antibodies against TMED10 (Cat# A305-228 A, 1:1000), PROM2 (Cat# TA500310, 1:1000), and GAPDH (Cat# AM4300, 1:5000) (all from Thermo Fisher Scientific). After washing, membranes were incubated with HRP-conjugated secondary antibody (Cat# 31460, 1:5000) and developed using the SuperSignal™ West Pico PLUS substrate. Bands were visualized using the ChemiDoc™ imaging system and quantified with ImageJ software.

For the cell proliferation assay, transfected A2780 and OVCAR3 cells were seeded into 96-well plates (5,000 cells/well), and viability was assessed at 24, 48, and 72 h using the Cell Counting Kit-8 (CCK-8, Dojindo, Japan). Absorbance was measured at 450 nm using a SpectraMax iD3 Multi-Mode Microplate Reader (Molecular Devices, USA), and the relative proliferation rate was calculated as a percentage of the control.

Colony formation assays were performed by seeding 500 transfected cells per well in 6-well culture plates (Thermo Fisher Scientific) and incubating them under standard culture conditions for 10–14 days to allow colony development [35, 36]. At the end of the incubation period, colonies were fixed with 4% paraformaldehyde (Thermo Fisher Scientific, Cat# 28908) for 15 min and then stained with 0.1% crystal violet solution (Thermo Fisher Scientific, Cat# Roti^®^-Histokit) for 20 min at room temperature. Plates were rinsed gently with distilled water to remove excess dye and air-dried. Colonies containing ≥ 40 cells were counted manually under a light microscope (Nikon Eclipse). All experiments were conducted in biological triplicates.

For the wound healing assay, transfected cells were seeded in 6-well plates (Thermo Fisher Scientific) and cultured to 90–100% confluence [37, 38]. A uniform linear scratch was created in the cell monolayer using a sterile 200 µL pipette tip. The wells were then gently washed with phosphate-buffered saline (PBS) (Thermo Fisher Scientific, Cat# 10010023) to remove detached cells, and the medium was replaced with serum-free RPMI-1640 medium (Thermo Fisher Scientific, Cat# 11875093) to minimize proliferation during migration assessment. Wound closure was monitored at 0 and 24 h, and images were captured using a phase-contrast microscope. The migration rate was calculated as the percentage of wound closure, defined as the reduction in wound width relative to the initial gap at 0 h, using ImageJ software (NIH, Bethesda, MD).

### Statistical analysis

All statistical analyses were conducted using R software (v4.2.0) or GraphPad Prism (v9.0). Differential gene expression was evaluated using the limma package with Benjamini–Hochberg correction; adjusted *p* < 0.05 was considered significant. RT-qPCR, proliferation, colony formation, and wound healing assays were analyzed using unpaired two-tailed t-tests. For immune subtype and drug sensitivity comparisons, Kruskal–Wallis and Spearman correlation tests were applied, respectively. Data are presented as mean ± standard deviation (SD), and p* < 0.05, p** < 0.01, and p*** < 0.01 were considered statistically significant unless otherwise specified. All experiments were performed in biological triplicates.

## Results

### Identification and validation of hub genes in OC

**Fig. 1:**
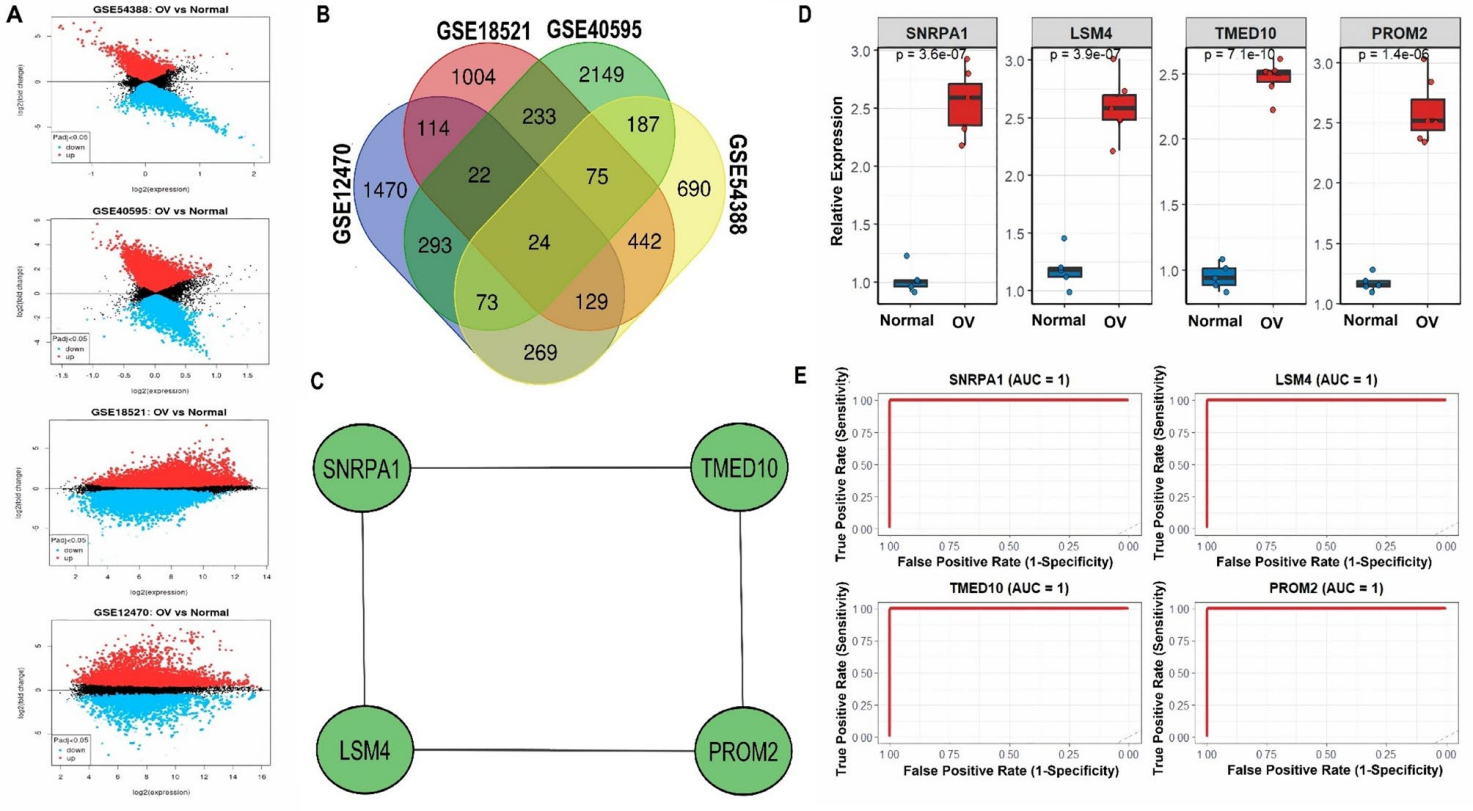
Identification and validation of hub genes in ovarian cancer (OC). (**A**) Volcano plots displaying differentially expressed genes (adjusted *p* < 0.05) in OC versus healthy tissues across four GEO datasets: GSE54388, GSE40595, GSE18521, and GSE12470. (**B**) Venn diagram showing 22 overlapping differentially expressed genes identified across the four datasets. (**C**) Protein–protein interaction (PPI) network of four selected hub genes—SNRPA1, LSM4, TMED10, and PROM2—based on their centrality. (**D**) RT-qPCR validation of hub gene expression in OC cell lines compared to healthy ovarian epithelial cell lines. All four genes show significant overexpression in OC cells (*p* < 0.00001). (**E**) Receiver operating characteristic (ROC) curves for the diagnostic evaluation of the four hub genes, each demonstrating excellent discriminatory power with an AUC of 1.0

To identify key genes involved in OC, we retrieved and analyzed four publicly available microarray expression datasets (GSE54388, GSE40595, GSE18521, and GSE12470) from the GEO database. Differential expression analysis between ovarian cancer and healthy tissue samples was performed using the limma package in R. The volcano plots presented in Fig. 1A illustrate the distribution of significantly (*p* < 0.05) upregulated and downregulated genes (adjusted *p* < 0.05) across each dataset (Fig. 1A). To uncover robust and reproducible gene expression signatures, we next performed intersectional analysis across the four datasets. A Venn diagram (Fig. 1B) was used to systematically identify genes consistently dysregulated in all datasets. This integrative approach revealed 22 genes that were commonly dysregulated in OC samples compared to healthy tissues (Fig. 1B). Among these, SNRPA1, LSM4, TMED10, and PROM2 hub genes were selected for further investigation based on their centrality in protein–protein interaction (PPI) networks, biological relevance, and consistent upregulation across datasets. The simplified PPI network presented in Fig. 1C depicts the interaction landscape among these four hub genes. To validate the expression patterns observed in silico, we performed RT-qPCR analysis in OC cell lines compared to healthy ovarian epithelial control cell lines. As shown in Fig. 1D, the expression of SNRPA1, LSM4, TMED10, and PROM2 was significantly (*p* < 0.05) upregulated in OC cells relative to healthy controls (*p* < 0.00001 for all genes) (Fig. 1D). To assess the diagnostic potential of these hub genes, ROC curve analysis was conducted. Remarkably, all four genes demonstrated excellent discriminatory power, with an area under the curve (AUC) of 1.0, indicating perfect sensitivity and specificity in distinguishing OC samples from healthy controls (Fig. 1E).

### Validation of hub gene expression in TCGA-OC cohort and functional pathway analysis

**Fig. 2:**
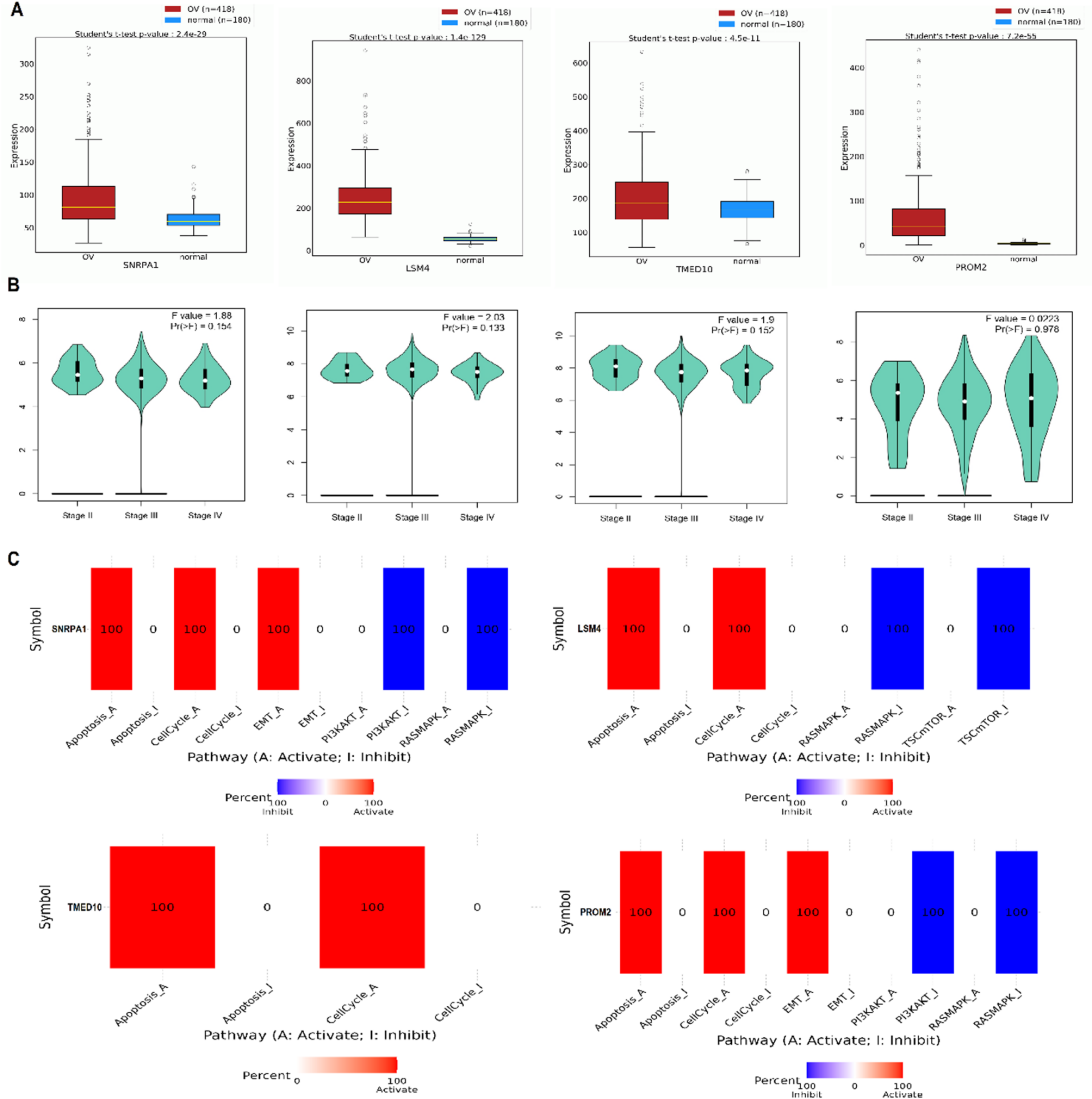
Expression and pathway activity of hub genes in the TCGA-OV cohort. (**A**) Box plots from GEPIA2 showing significantly higher expression of SNRPA1, LSM4, TMED10, and PROM2 in ovarian cancer tissues (*n* = 419) compared to healthy tissues (*n* = 88). (**B**) Violin plots depicting expression levels of hub genes across stages II–IV of OC. No statistically significant variation in gene expression across stages was observed (*p* > 0.05). (**C**) Pathway activity analysis from GSCA showing positive correlation of hub genes with oncogenic pathways (e.g., apoptosis, cell cycle, EMT) and negative correlation with tumor-suppressive pathways (e.g., RAS/MAPK, PI3K/AKT)

To further validate the differential expression of the identified hub genes, we utilized RNA-seq data from TCGA OV cohort via the GEPIA2 platform. As illustrated in Fig. 2A, all four hub genes—SNRPA1, LSM4, TMED10, and PROM2—exhibited significantly (*p* < 0.05) elevated expression in OC tissues (*n* = 419) compared to healthy ovarian tissues (*n* = 88). Specifically, SNRPA1 (*p* = 1.4e–29), LSM4 (*p* = 1.3e–29), TMED10 (*p* = 4.5e–31), and PROM2 (*p* = 2.2e–25) demonstrated strong overexpression, in line with our prior analyses from GEO datasets and RT-qPCR validations (Fig. 2A). We next examined whether the expression levels of these hub genes were associated with disease stage progression in OC. As shown in Fig. 2B, violin plots from GEPIA2 revealed that while the expression of SNRPA1, LSM4, TMED10, and PROM2 was consistently high across stages II, III, and IV, the differences between stages were not statistically significant (all *p* > 0.05) (Fig. 2B), suggesting that while these genes are dysregulated in cancer, their expression may not vary markedly with clinical stage. To gain insight into the potential functional roles of the identified hub genes in OV, we performed pathway activity analysis using the GSCA platform. The results are shown in Fig. 2C, where we observed that SNRPA1, LSM4, TMED10, and PROM2 were all positively associated with the activation of key oncogenic pathways, including apoptosis, cell cycle progression, epithelial–mesenchymal transition (EMT), and DNA damage response (Fig. 2C). Conversely, these genes were found to inhibit several tumor-suppressive signaling pathways, such as the RAS/MAPK and PI3K/AKT pathways (Fig. 2C).

### Promoter methylation and meta-analysis-based survival evaluation of hub genes

**Fig. 3:**
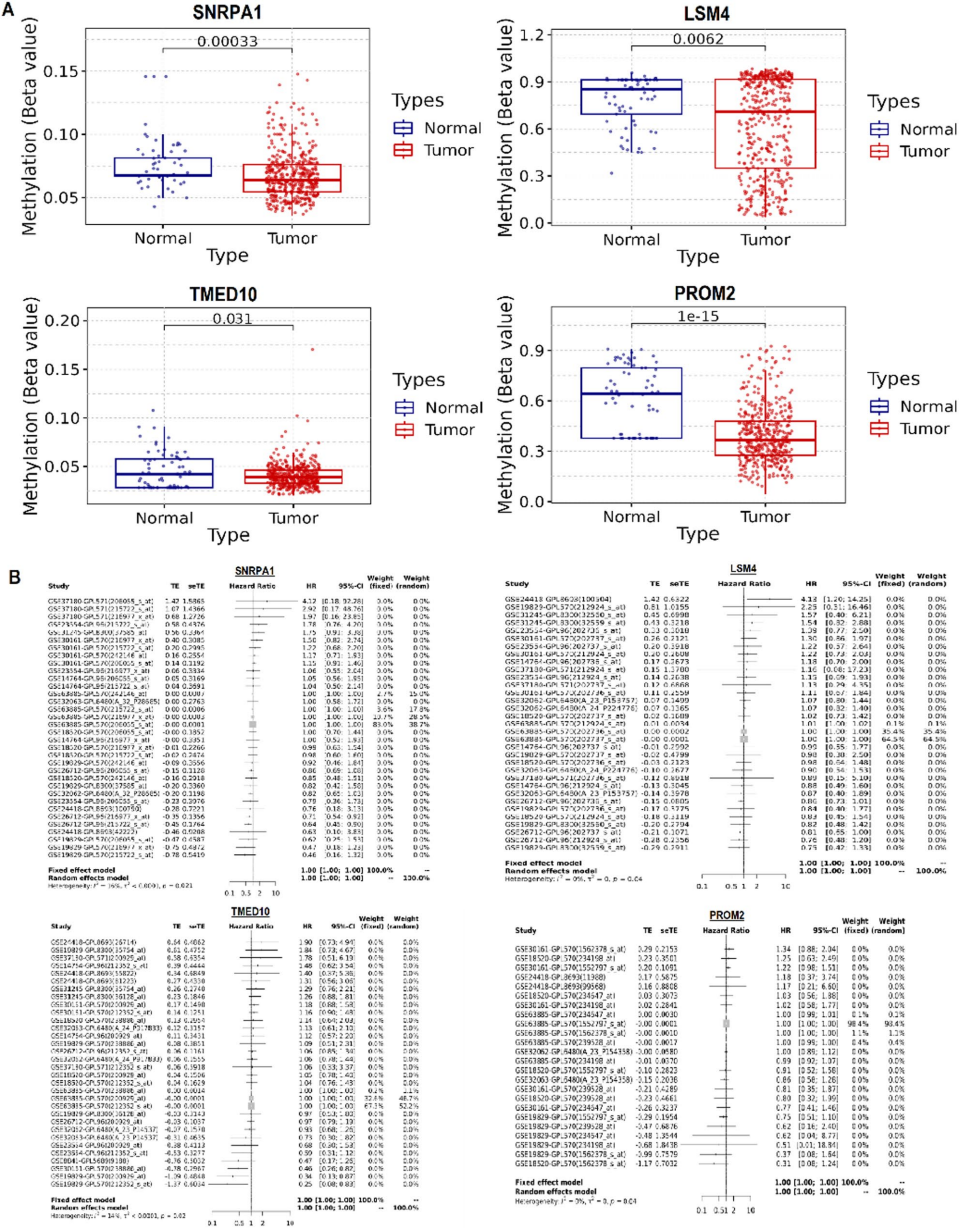
Promoter methylation and survival analysis of hub genes in ovarian cancer (OC). (**A**) Promoter methylation status of hub genes in OC, assessed via GSCA. (**B**) Forest plots from GENT2 meta-analysis summarizing hazard ratios (HRs) and 95% confidence intervals for each hub gene across multiple studies. P-value < 0.05

To investigate the potential epigenetic regulation of the identified hub genes, we examined their promoter methylation status in OC using the GSCA platform, which integrates TCGA methylation data. As depicted in Fig. 3A, all four hub genes—SNRPA1, LSM4, TMED10, and PROM2—exhibited significantly reduced promoter methylation levels in tumor tissues compared to healthy ovarian tissues (Fig. 3A). Specifically, methylation beta values were significantly lower for SNRPA1 (*p* = 0.00033), LSM4 (*p* = 0.0062), TMED10 (*p* = 0.031), and PROM2 (*p* = 1e–15) (Fig. 3A). To evaluate the prognostic significance of the hub genes, we performed a meta-analysis of survival data using the GENT2 database, which aggregates gene expression and clinical outcome data across multiple independent GEO datasets. As shown in Fig. 3B, forest plots illustrate the hazard ratios (HRs) and 95% confidence intervals for each gene across individual studies (Fig. 3B). The fixed-effect models for all four genes—SNRPA1, LSM4, TMED10, and PROM2—yielded pooled HRs centered around 1.00, indicating no significant (*p* > 0.05) association between their expression and overall survival in OC patients (Fig. 3B).

### Mutational and CNV analysis of hub genes in OC

**Fig. 4:**
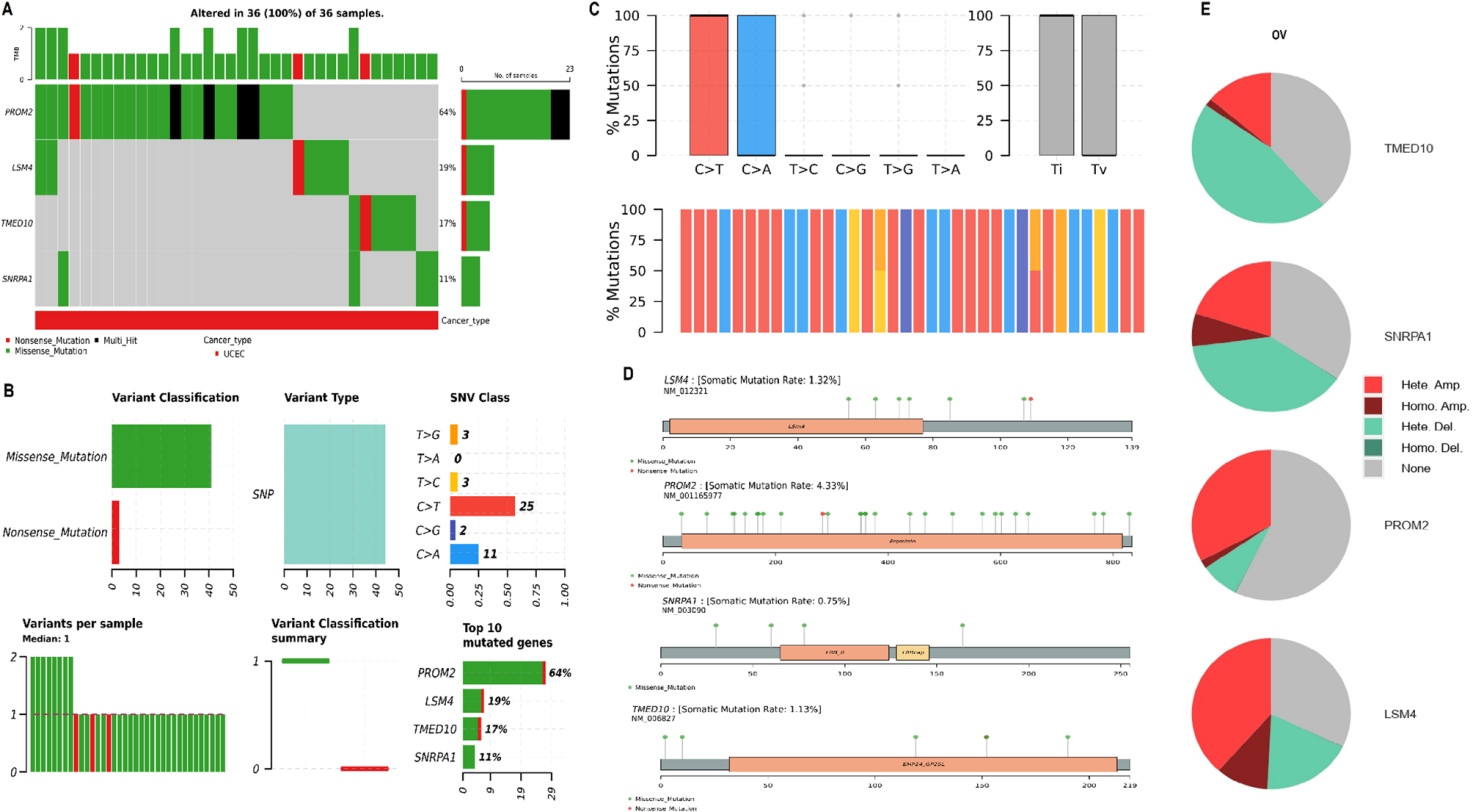
Mutational and copy number variation (CNV) analysis of hub genes. (**A**) Mutation frequency and distribution of SNRPA1, LSM4, TMED10, and PROM2 in OC samples from cBioPortal. (**B**) Classification of mutation types and SNV classes. (**C**) Transition and transversion (Ti/Tv) rates showing Ti predominance in the mutational spectrum. (**D**) Domain-level mapping of mutations indicating potential functional disruption across key domains of the hub proteins. (**E**) GISTIC-based CNV analysis showing prevalence of heterozygous amplifications and deletions in the hub genes across OC samples

To further elucidate the genomic alterations associated with the identified hub genes in OC, we performed comprehensive mutational profiling and (CNV analysis using the cBioPortal platform. As illustrated in Fig. 4A, all four hub genes—PROM2, LSM4, TMED10, and SNRPA1—exhibited somatic mutations across all 36 analyzed OC samples (100%). PROM2 was the most frequently mutated gene (64%), followed by LSM4 (19%), TMED10 (17%), and SNRPA1 (11%). The most prevalent mutation types were missense mutations, with occasional nonsense and multi-hit variants (Fig. 4A). Variant classification and substitution frequency are summarized in Fig. 4B. The predominant variant classification was missense mutations, and single nucleotide polymorphisms (SNPs) represented the major variant type (Fig. 4B). Notably, C > T transitions were the most frequent single nucleotide variant (SNV) class, followed by C > A substitutions (Fig. 4B). In Fig. 4C, transition (Ti) and transversion (Tv) rates further confirmed the dominance of Ti events, consistent with patterns commonly observed in solid tumors (Fig. 4B). Domain-level mapping of mutations in Fig. 4D indicates that mutations in LSM4, PROM2, TMED10, and SNRPA1 are dispersed across functional domains, potentially affecting protein function (Fig. 4D). CNV for hub genes were assessed in ovarian cancer samples using cBioPortal’s GISTIC-based CNV data (Fig. 4E). The analysis revealed that heterozygous amplifications (Hete. Amp.) and heterozygous deletions (Hete. Del.) were the most common forms of CNV observed across hub genes (Fig. 4E).

### PPI network and gene enrichment analysis of hub genes

**Fig. 5:**
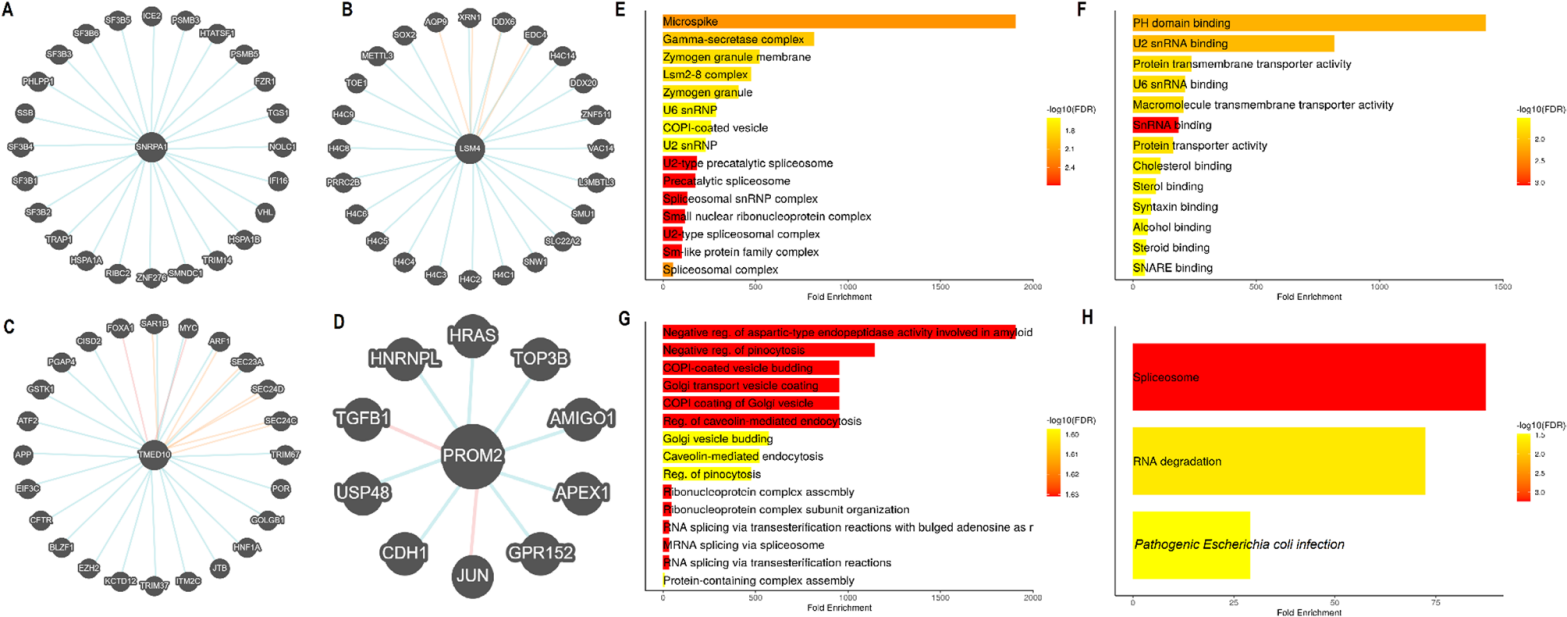
PPI networks and functional enrichment of hub genes. (**A**–**D**) Protein–protein interaction networks for SNRPA1, LSM4, TMED10, and PROM2, generated using the Pathway Commons database. (**E**) GO cellular component enrichment. (**F**-**G**) GO molecular function and biological process enrichment. (**H**) KEGG pathway enrichment analysis showing enrichment in spliceosome, RNA degradation, and pathogenic *E. coli* infection pathways. P-value < 0.05

To further elucidate the molecular functions and biological roles of the identified hub genes in OC, we constructed PPI networks and performed comprehensive functional enrichment analyses. The PPI networks for each hub gene—SNRPA1, LSM4, TMED10, and PROM2—were generated using the Pathway Commons database (Fig. 5A-D). The interaction networks of SNRPA1, LSM4, TMED10, and PROM2 revealed multiple interacting partners. Blue lines represented direct binding interactions, pink lines indicated co-expression-based associations, orange lines denoted modification-related partners, and grey lines illustrated other types of interactions (Fig. 5A–D). To explore the biological functions of the hub gene-interacting partners, gene ontology (GO) and Kyoto Encyclopedia of Genes and Genomes (KEGG) enrichment analyses were conducted using the DAVID database. GO analysis revealed that in the cellular component (CC) category, the binding partners of hub genes were significantly enriched in spliceosomal complexes, U2-type snRNPs, and COPI-coated vesicles, supporting their known roles in RNA processing and intracellular transport (Fig. 5E). In terms of molecular function (MF), the enriched terms included PH domain binding, U2 snRNA binding, and protein transmembrane transporter activity, indicating that the hub gene interactors are involved in nucleic acid binding and membrane trafficking processes (Fig. 5F). Biological process (BP) enrichment analysis further emphasized the role of these networks in vesicle-mediated transport, pinocytosis, and mRNA splicing, suggesting potential mechanistic links between the hub genes and altered RNA metabolism and endocytic activity in ovarian cancer cells (Fig. 5G). KEGG pathway analysis demonstrated that the binding partners of the hub genes were significantly enriched in pathways related to the spliceosome, RNA degradation, and pathogenic *Escherichia coli* infection (Fig. 5H).

### miRNA–hub gene interaction analysis

**Fig. 6:**
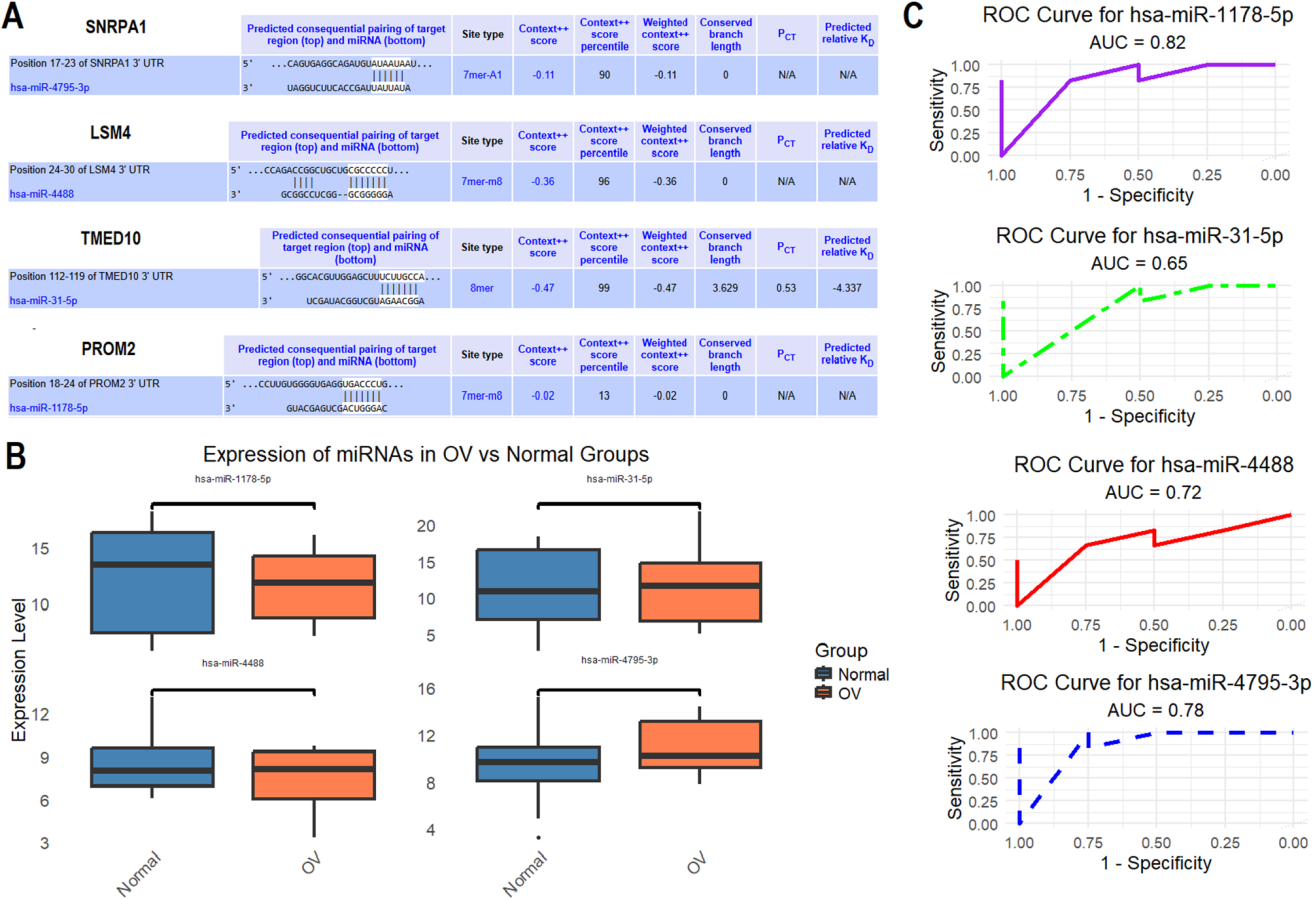
miRNA–hub gene regulatory network and diagnostic evaluation in ovarian cancer (OC). (**A**) Predicted miRNA–mRNA interactions for hub genes using the TargetScan database. (**B**) RT-qPCR analysis of miRNA expression in OC vs. healthy control cell lines, showing significant downregulation of all four candidate miRNAs in OC cells (*p* < 0.001). (**C**) ROC curve analyses evaluating diagnostic performance of miRNAs. P-value < 0.05

To explore the post-transcriptional regulatory mechanisms governing the expression of the identified hub genes in OC, we performed miRNA target prediction, expression profiling, and diagnostic performance evaluation. In Fig. 6A, the TargetScan database was employed to predict potential miRNAs that may bind to the 3’ untranslated regions (3’ UTRs) of the hub genes—SNRPA1, LSM4, TMED10, and PROM2. The prediction results revealed strong putative interactions, including hsa-miR-4795-3p targeting SNRPA1 at position 17–23, hsa-miR-4488 targeting LSM4 at position 24–30, hsa-miR-31-5p targeting TMED10 at position 112–119, and hsa-miR-1178-5p targeting PROM2 at position 18–24 (Fig. 6A). Next, to validate the expression of these candidate regulatory miRNAs, we performed RT-qPCR analysis using OC and healthy control cell lines (Fig. 6B). The results demonstrated significant (*p* < 0.05) downregulation of all four miRNAs in OC samples compared to the healthy group. To evaluate the diagnostic utility of these miRNAs in distinguishing ovarian cancer from healthy samples, ROC curve analyses were conducted (Fig. 6C). hsa-miR-1178-5p exhibited the highest diagnostic performance with an AUC of 0.82, followed by hsa-miR-4795-3p (AUC = 0.78), hsa-miR-4488 (AUC = 0.72), and hsa-miR-31-5p (AUC = 0.65) (Fig. 6C).

### Immunological and therapeutic potential of hub genes

**Fig. 7:**
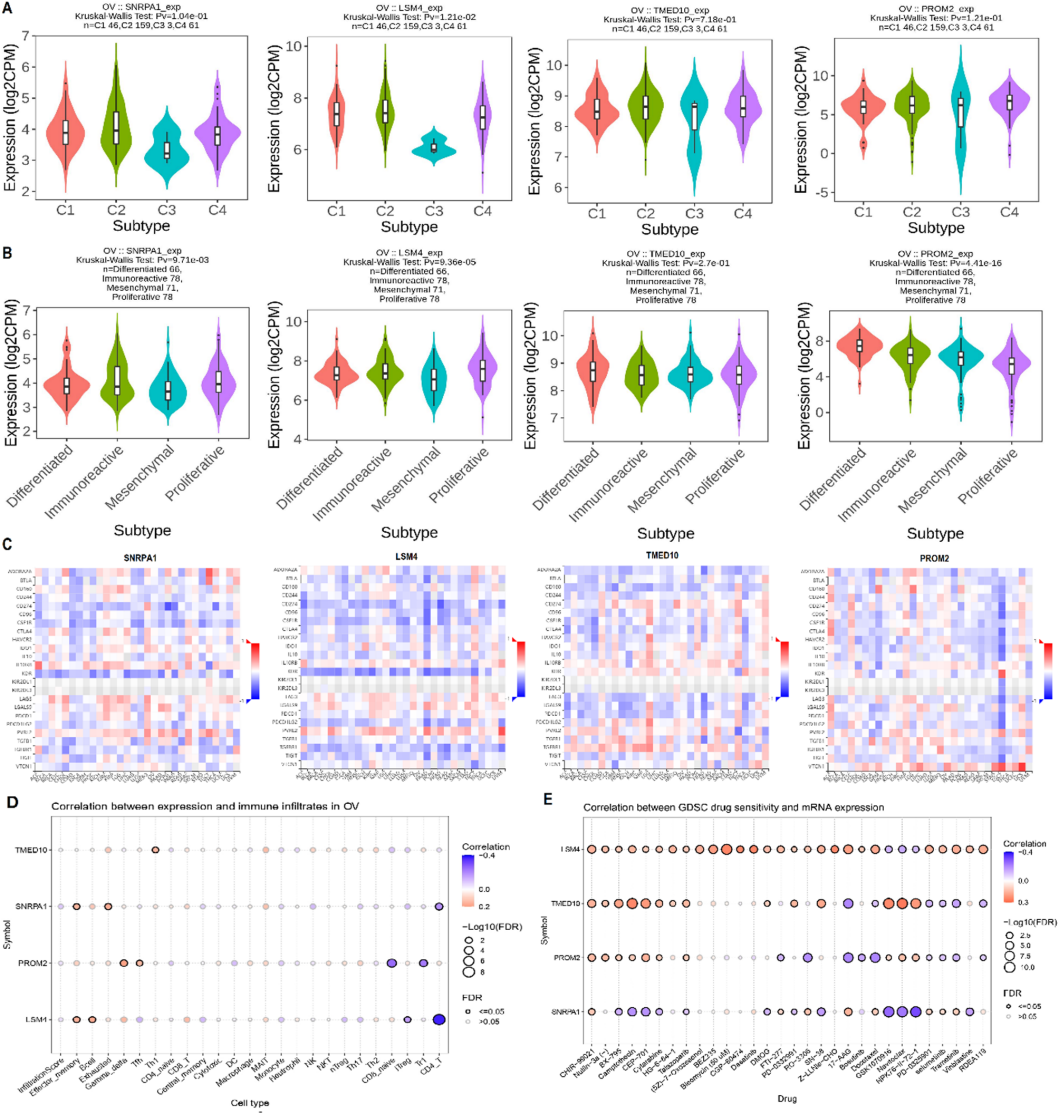
Immunological and therapeutic significance of hub genes in ovarian cancer (OC). (**A**) Expression of hub genes across OC immune subtypes based on TCGA data using TISIDB. (**B**) Differential expression of hub genes among molecular subtypes of OC. (**C**) Heatmap showing positive correlations between hub gene expression and immune inhibitory genes, including strong associations of SNRPA1 with VTCN1 and IDO1. (**D**) Correlation heatmap between hub gene expression and immune cell infiltration, highlighting negative correlations with Th1 and MAIT cells for SNRPA1 and TMED10. (**E**) Drug sensitivity correlation analysis from GDSC. P-value < 0.05

To further explore the immunological and therapeutic relevance of the identified hub genes (SNRPA1, LSM4, TMED10, and PROM2) in OC, we conducted a series of in silico analyses using expression-based and drug sensitivity datasets. In Fig. 7A, the expression profiles of these hub genes were evaluated across different OC immune subtypes using the TCGA dataset using TISIDB database. The results revealed statistically significant expression variation among subtypes C1 to C4 for all four genes (Kruskal–Wallis test, *p* < 0.01) (Fig. 7A). Figure 7B presents the expression patterns of the hub genes across molecular subtypes of OC, specifically Differentiated, Immunoreactive, Mesenchymal, and Proliferative classes. All four genes demonstrated significant differential expression across subtypes (*p* < 0.001) (Fig. 7B). In Fig. 7C, the correlation heatmaps between hub genes and immune inhibitor genes were generated using TISIDB database to examine their immunomodulatory potential. A predominance of red-colored tiles indicates a strong positive correlation, particularly between SNRPA1 and immune checkpoint inhibitors such as VTCN1 and IDO1 (Fig. 7C). Similarly, PROM2 and TMED10 showed moderate to strong positive associations with several immunosuppressive genes (Fig. 4C). Figure 7D explores the correlation between hub gene expression and immune cell infiltration levels. Here, the blue-colored tiles represent significant negative correlations, highlighting the potential immune-suppressive effect of high hub gene expression. For instance, SNRPA1 and TMED10 expression showed consistent negative correlations with Th1 cells and MAIT cells indicating that their overexpression may hinder immune cell recruitment or function within the tumor microenvironment (Fig. 7D). Figure 7E illustrates the correlation between gene expression and sensitivity to various chemotherapeutic agents using the GDSC database. Red-colored circles in this panel signify positive correlations, with PROM2 and TMED10 exhibiting strong positive associations with multiple drugs, including Vinblastine, BX-795, and SN-38. This suggests that elevated levels of the hub genes may confer drug resistance in OC cells. Incorporating IC₅₀ correlations adds important translational value, highlighting the potential of hub genes as biomarkers for predicting resistance to specific chemotherapeutic agents.

### TMED10 and PROM2 knockdown suppresses proliferation, clonogenicity, and migration in OC cells

**Fig. 8:**
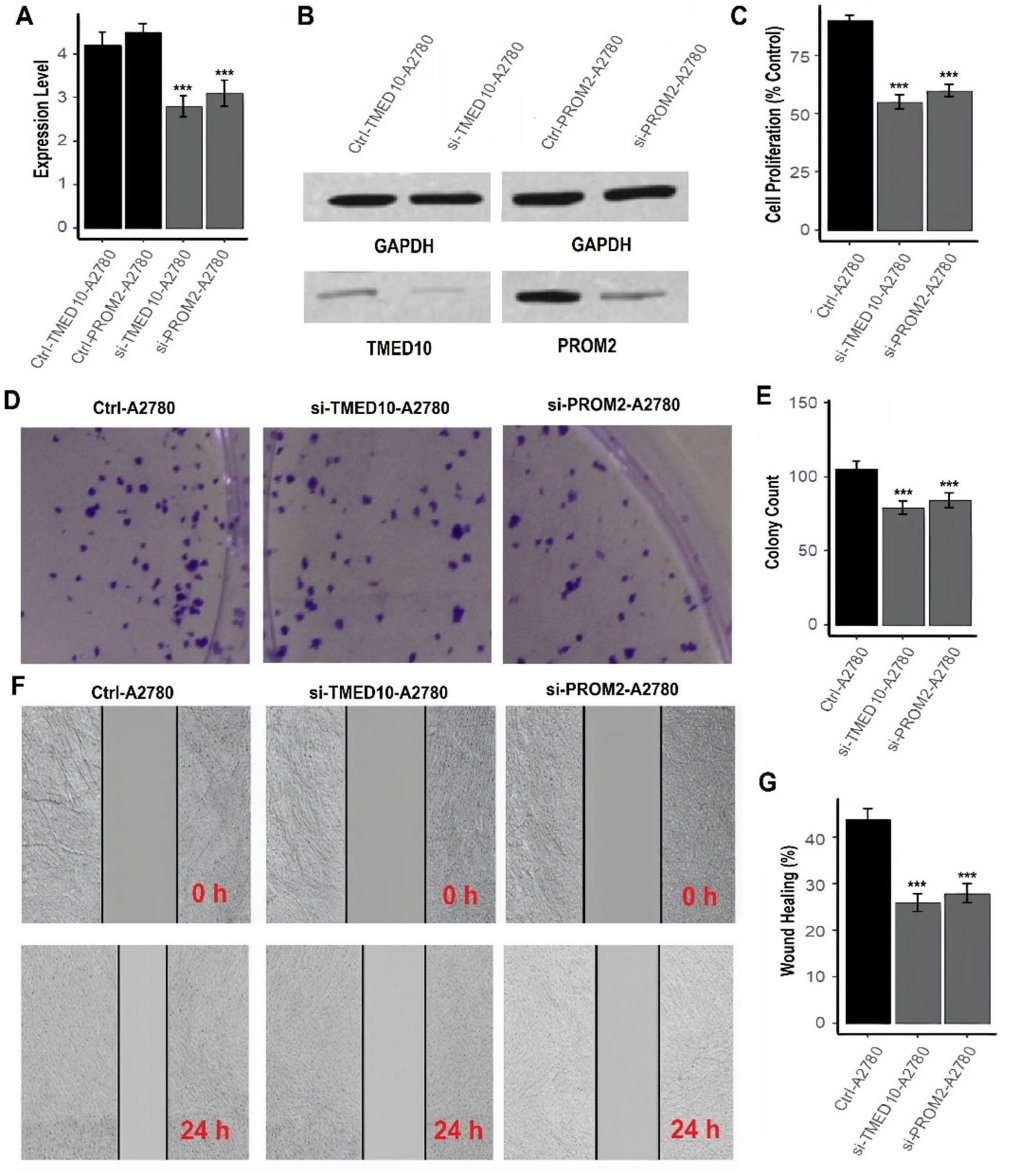
TMED10 knockdown suppresses OC cell proliferation, clonogenicity, and migration in A2780 cells. (**A**) RT-qPCR confirms TMED10 knockdown efficiency in A2780 cells (p*** < 0.001). (**B**) Western blot validation of TMED10 knockdown at protein level. (**C**) CCK-8 assay reveals reduced proliferation following TMED10 silencing (~ 55–60% of control; p*** < 0.001). (D–E) Colony formation assay shows decreased clonogenic ability post-knockdown. (F–G) Wound healing assay demonstrates impaired migratory capacity of TMED10-silenced A2780 cells

**Fig. 9:**
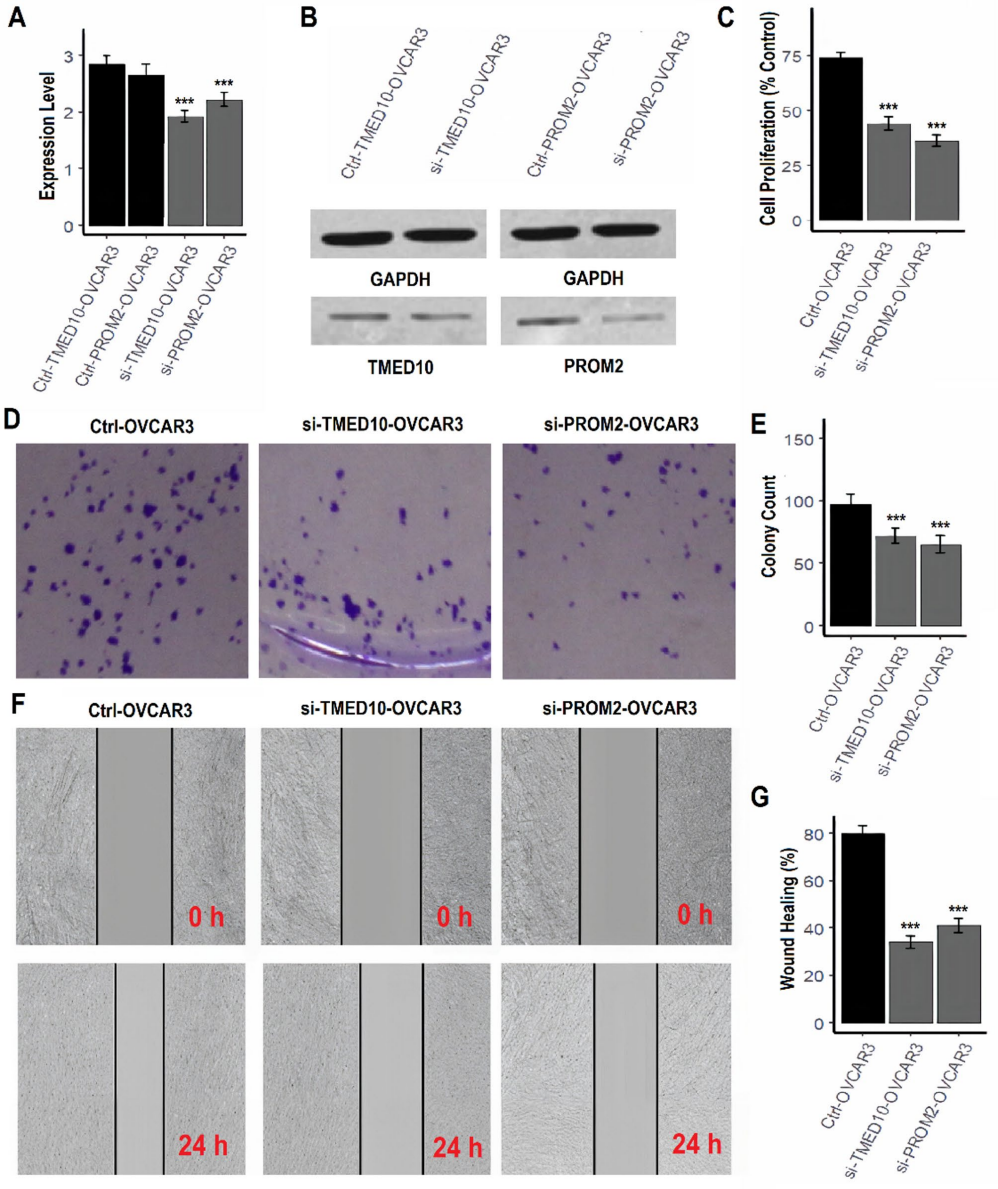
PROM2 knockdown suppresses OC cell proliferation, clonogenicity, and migration in OVCAR3 cells. (**A**) RT-qPCR validation of PROM2 knockdown in OVCAR3 cells (p*** < 0.001). (**B**) Western blot confirms reduced PROM2 protein expression post-siRNA treatment. (**C**) Cell proliferation significantly reduced after PROM2 knockdown (p*** < 0.001). (**D**–**E**) Colony formation assay indicates a marked decrease in long-term survival potential. (**F**–**G**) Wound healing assay reveals significantly impaired cell motility after PROM2 silencing

To investigate the functional roles of TMED10 and PROM2 in OC, we performed siRNA-mediated knockdown of these genes in two human OC cell lines, A2780 and OVCAR3, followed by multiple in vitro assays. RT-qPCR analysis confirmed a significant reduction in TMED10 and PROM2 mRNA levels in both A2780 and OVCAR3 cells transfected with respective siRNAs compared to control cells (Figs. 8A and 9A, ***P < 0.001). Western blotting further validated the knockdown at the protein level (Figs. 8B and 9B, supplementary data Fig. 1), demonstrating efficient silencing of both targets. CCK-8 cell proliferation assays revealed that knockdown of TMED10 or PROM2 significantly reduced cell viability in both A2780 (Fig. 8C) and OVCAR3 (Fig. 9C) cell lines. Quantitatively, TMED10 and PROM2 silencing reduced proliferation to approximately 55–60% of the control levels (***P < 0.001), indicating a strong inhibitory effect on cancer cell growth. Colony formation assays demonstrated that the clonogenic potential of A2780 and OVCAR3 cells was markedly reduced following siRNA-mediated knockdown of TMED10 or PROM2 (Figs. 8D–E and 9D–E). The number of colonies was significantly decreased in the knockdown groups compared to controls (***P < 0.001), suggesting a critical role for these genes in sustaining long-term proliferative capacity. To further assess the effects of TMED10 and PROM2 knockdown on cell motility, wound healing assays were performed. In both A2780 and OVCAR3 cells, TMED10 and PROM2 silencing led to a marked decrease in wound closure after 24 h compared to control groups (Figs. 8F–G and 9F–G). Quantification showed a significant reduction in wound healing percentages (***P < 0.001), indicating impaired migratory potential upon gene silencing.

**Fig. 10:**
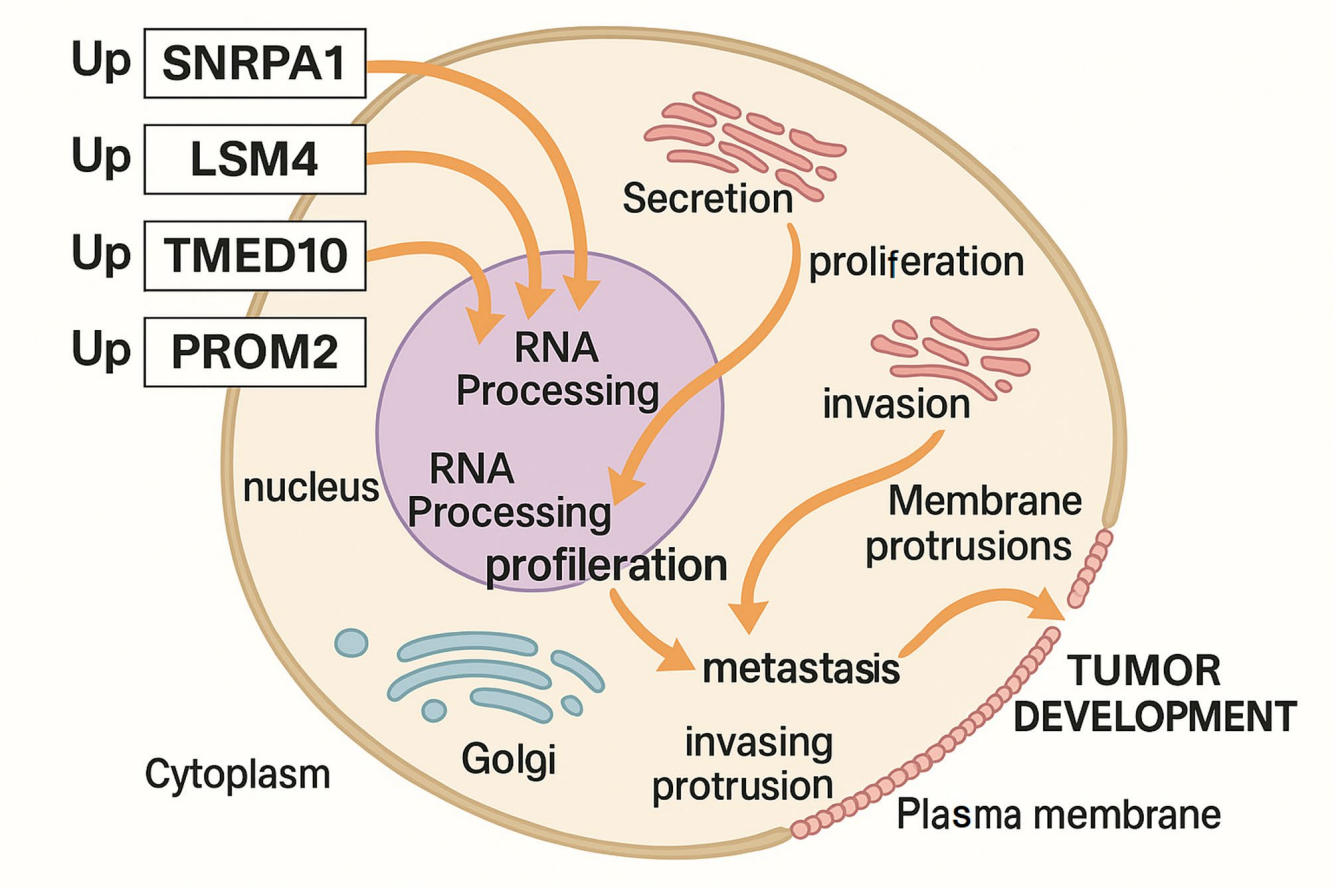
Proposed mechanistic model of hub gene–mediated ovarian cancer (OC) progression. Schematic representation illustrating the roles of SNRPA1, LSM4, TMED10, and PROM2 in promoting OC progression

Finally, based on the integration of transcriptomic analysis and functional assays, we propose a mechanistic model illustrating how upregulation of SNRPA1, LSM4, TMED10, and PROM2 contributes to OC progression (Fig. 10). All four genes were found to be significantly upregulated in cancer cells and are implicated in promoting multiple cancer hallmarks through distinct but interconnected cellular processes. SNRPA1 and LSM4, which are core components of the spliceosomal and RNA-binding machinery, appear to drive aberrant RNA processing, which in turn may dysregulate the expression of key oncogenes and tumor suppressors (Fig. 10). This aberrant RNA processing contributes to enhanced proliferation and may facilitate epithelial-mesenchymal transition (EMT), leading to increased invasion and metastasis. TMED10, a member of the p24 cargo receptor family, is involved in protein trafficking and Golgi processing (Fig. 10). Its upregulation enhances secretion and membrane protrusion formation, both of which are crucial for cell migration and invasion (Fig. 10). PROM2, a membrane-associated protein, contributes to vesicular trafficking and plasma membrane remodeling, supporting cell motility, metastatic dissemination, and possibly chemoresistance through the regulation of proliferative signaling at the membrane level (Fig. 10).

## Discussion

OC remains one of the deadliest gynecologic malignancies, largely due to late-stage diagnosis, frequent recurrence, and development of chemoresistance [1, 39, 40]. Although several molecular alterations have been implicated in OC pathogenesis, the precise regulatory networks that govern disease progression remain incompletely understood [41–43]. In this study, we identified four consistently upregulated hub genes—SNRPA1, LSM4, TMED10, and PROM2—through integrated bioinformatics analysis of multiple transcriptomic datasets, validated their expression and function in vitro, and proposed a mechanistic model linking them to RNA metabolism, membrane trafficking, and oncogenic behavior in OC cells.

While prior studies have implicated SNRPA1 and LSM4 in RNA splicing and cancer [44, 45], and TMED10 in autophagy and vesicle trafficking in melanoma and liver cancer [46, 47], their involvement in OC has been explored systematically by a limited number of studies. Notably, Hou et al. demonstrated that circ_0025033 promotes OC progression by upregulating LSM4 through sponging miR-184. Functional validation revealed that knockdown of either circ_0025033 or LSM4 significantly inhibited colony formation, migration/invasion, and glycolysis in OC cell lines, implicating LSM4 as a key driver of tumor growth and metabolic reprogramming [48]. Additionally, Wang et al. performed an integrated bulk and single-cell RNA-seq analysis, identifying LSM4 as part of a prognostic signature in OC. They reported that LSM4 was upregulated in ovarian tumors and exhibited dynamic expression across epithelial, endothelial, and stromal cell types, including fibroblasts, during differentiation processes [49].

PROM2 has previously been associated with chemoresistance and membrane remodeling in triple-negative breast cancer [50], but a few studies to date has characterized its expression or function in OC. Notably, Li et al. showed that artesunate (ART) induces ferroptosis and suppresses OC progression by inhibiting HOXC11, which transcriptionally regulates PROM2 [51]. In that study, HOXC11 overexpression rescued ART-induced effects on proliferation, migration, apoptosis, and ferroptosis by activating PROM2/PI3K/AKT signaling, whereas PROM2 silencing restored sensitivity to ART and tumor suppression both in vitro and in vivo [51]. Additionally, Saha et al. conducted a multi‑omics analysis across cancers and found that PROM2, along with PROM1, differentially modulates prognosis: PROM2 overexpression was associated with poor clinical outcomes in several tumor types, including OC [52]. Our validation by RT-qPCR and ROC curve analyses confirmed not only the overexpression of these genes but also their near-perfect diagnostic value (AUC = 1.0), underscoring their clinical relevance.

Using TCGA RNA-seq data, we confirmed significant overexpression of all four genes in OC tissue compared to healthy ovary tissues. Notably, while their expression remained high across stages II–IV, no significant difference was observed across stages. This finding aligns with previous studies showing that certain oncogenes (e.g., BUB1B, MELK) maintain high expression throughout OC progression without stage specificity [53, 54]. Pathway activity analysis revealed that all hub genes were associated with activation of cancer hallmark pathways, including cell cycle progression, EMT, and DNA damage response. These results are consistent with prior findings implicating SNRPA1 and LSM4 in transcriptional regulation and chromatin remodeling [55, 56], and TMED10 and PROM2 in vesicle trafficking and cell polarity [35, 57, 58]. Importantly, our study adds novel evidence linking these genes with suppression of tumor-suppressive pathways like PI3K/AKT and RAS/MAPK, which has not been previously reported.

We further explored the epigenetic regulation of these hub genes and found significantly reduced promoter methylation in OC samples. Hypomethylation has been widely recognized as a mechanism of oncogene activation in various cancers, including OC [59, 60]. Our findings support this model and suggest that epigenetic derepression may be a key mechanism contributing to overexpression of SNRPA1, LSM4, TMED10, and PROM2. However, meta-analysis using the GENT2 database did not reveal significant associations between hub gene expression and patient survival. This contrasts with prior reports linking SNRPA1 and LSM4 expression to poor prognosis in colorectal and liver cancers [61, 62], highlighting possible tissue-specific roles or the influence of other clinical and molecular confounders in OC.

Functional enrichment of hub gene-interacting partners revealed strong associations with spliceosomal complexes, transmembrane transporters, and vesicle-mediated trafficking pathways. This supports existing literature on the roles of SNRPA1 and LSM4 in RNA splicing and stability [62, 63], and TMED10 and PROM2 in protein secretion and Golgi function [64, 65]. Notably, KEGG pathway analysis also highlighted enrichment in RNA degradation and bacterial infection pathways, pointing toward possible immune-modulatory roles that merit further investigation. These enrichment patterns collectively reinforce the idea that dysregulated RNA processing and intracellular trafficking are central to OC progression.

We also identified miRNAs that potentially target the 3′ UTRs of the four hub genes and demonstrated their significant downregulation in OC cells. This inverse correlation supports their role in post-transcriptional regulation. Several of these miRNAs (e.g., hsa-miR-31-5p, hsa-miR-1178-5p) have been implicated in tumor suppression and immune modulation [66, 67]. Our ROC analysis revealed that these miRNAs, especially hsa-miR-1178-5p, exhibit reasonable diagnostic potential (AUC = 0.82), suggesting that a combined mRNA–miRNA signature could enhance diagnostic accuracy in OC.

In silico immune landscape analysis revealed significant associations of hub genes with immune checkpoint genes and immune cell infiltration. The strong positive correlation of SNRPA1, TMED10, and PROM2 with suppressive immune regulators like IDO1 and VTCN1, and negative correlation with Th1 and MAIT cell infiltration, suggest that these genes may contribute to an immune-evasive tumor microenvironment. This observation complements earlier findings showing that splicing-related factors can modulate antigen presentation and immune escape [68–70].

Finally, siRNA-mediated knockdown of TMED10 and PROM2 in A2780 and OVCAR3 cells resulted in significant reductions in proliferation, colony formation, and migration. These findings are in line with previous functional studies in liver and breast cancer models, where TMED10 and PROM2 silencing suppressed tumor cell growth and invasion [71, 72]. Mechanistically, TMED10 may exert tumor-promoting effects via disruption of TGF-β receptor complex formation, as observed in lung cancer models, where it attenuates Smad2 activation and downstream TGF-β signaling pathways [73, 74]. PROM2, on the other hand, may facilitate oncogenic phenotypes by promoting PI3K/AKT pathway signaling [75], enhancing cell survival under therapeutic stress, as reported in prior chemoresistance models. Future studies should aim to delineate the downstream signaling pathways and intracellular trafficking networks governed by TMED10 and PROM2 in OC, including direct interactions with TGF-β signaling components and AKT pathway mediators. Moreover, evaluating their clinical utility for patient stratification—particularly in predicting response to platinum-based, taxane, or novel agents—will be critical for potential translation into precision oncology strategies.

## Conclusion

In conclusion, our comprehensive multi-omics analysis identifies SNRPA1, LSM4, TMED10, and PROM2 as key oncogenic drivers in OC, functioning through distinct yet converging mechanisms involving RNA processing, protein trafficking, and immune modulation. These genes serve as potential biomarkers for diagnosis and may represent novel therapeutic targets. Future studies are warranted to explore their roles in vivo, dissect their downstream effectors, and evaluate their utility in combination with immune or chemotherapeutic agents in preclinical models.

## Supplementary Information

Below is the link to the electronic supplementary material.

Supplementary Material 1

## Data Availability

Any type of the data will be provided by the corresponding author.
