# Supplementary figures and images for: Multi-Omics analysis and in vitro validation reveal diagnostic and therapeutic roles of novel hub genes in ovarian cancer
Source: Hereditas. 2025 Aug 18;162:166. doi: 10.1186/s41065-025-00535-z (PMC12363112; doi:10.1186/s41065-025-00535-z)

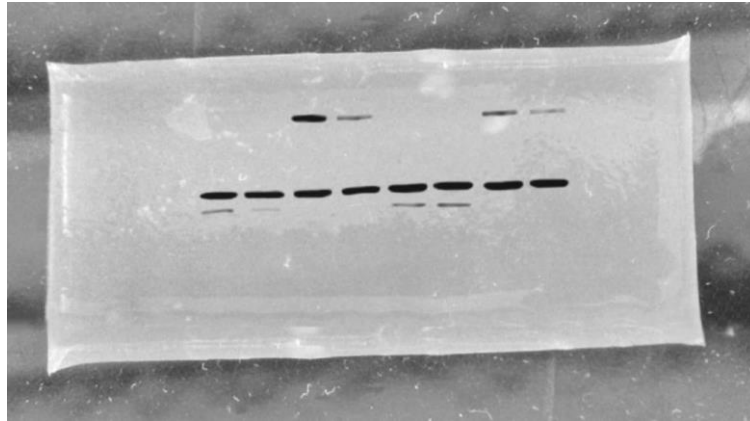

**Supplementary data Figure 1: Uncut Western blot bands of TMED10, PROM2, and GAPDH.**

Supplement: Supplementary file 1 — Supplementary Material 1 [file 41065_2025_535_MOESM1_ESM.pdf]
